# Playback of 50-kHz ultrasonic vocalizations overcomes psychomotor deficits induced by sub-chronic haloperidol treatment in rats

**DOI:** 10.1007/s00213-020-05517-9

**Published:** 2020-05-18

**Authors:** Liana Melo-Thomas, Luan C. Tonelli, Christian P. Müller, Markus Wöhr, Rainer K. W. Schwarting

**Affiliations:** 1 Experimental and Biological Psychology, Behavioral Neuroscience, Faculty of Psychology, Philipps-University of Marburg, Gutenbergstraße 18, 35032 Marburg, Germany; 2 Center for Mind, Brain, and Behavior (CMBB), Hans-Meerwein-Straße 6, 35032 Marburg, Germany; 3 Institute of Neuroscience and Behavior (INeC), Av. do Café, 2450, Monte Alegre, Ribeirão Preto, São Paulo 14050-220 Brazil; 4 Section of Addiction Medicine, Department of Psychiatry and Psychotherapy, University Clinic, Friedrich-Alexander-University Erlangen-Nuremberg, Erlangen, Germany

**Keywords:** Paradoxical kinesia, Ultrasonic vocalization, Bradykinesia, Haloperidol, Sub-chronic

## Abstract

**Rationale:**

In rodents, acute haloperidol treatment induces psychomotor impairments known as catalepsy, which models akinesia in humans and is characterized as an animal model of acute Parkinsonism, whereas sub-chronic haloperidol reduces exploratory behavior, which resembles bradykinesia. Haloperidol-induced catalepsy in rats can be ameliorated by playback of 50-kHz ultrasonic vocalizations (USV), an emotionally and motivationally relevant appetitive auditory stimulus, representing an animal model of paradoxical kinesia. In a condition like PD where patients suffer from chronic motor impairments, it is paramount to assess the long-term symptom relief in an animal model of Parkinsonism.

**Objectives:**

We investigated whether 50-kHz USV playback ameliorates psychomotor deficits induced by haloperidol in a sub-chronic dosing regimen.

**Methods:**

In phase 1, distance traveled and number of rearing behavior were assessed in an activity chamber in order to investigate whether sub-chronic haloperidol treatment induced psychomotor impairments. In phase 2, we investigated whether 50-kHz USV playback could overcome these impairments by assessing exploratory behaviors and approach behavior towards the sound source in the 50-kHz USV radial maze playback paradigm.

**Results:**

Sub-chronic haloperidol treatment led to psychomotor deficits since the distance traveled and number of rearing behavior were reduced as compared to saline control group or baseline. These psychomotor impairments were ameliorated during playback of 50-kHz USV, with haloperidol treated rats showing a clear social approach behavior towards the sound source exclusively during playback.

**Conclusions:**

This study provides evidence that 50-kHz USV playback induces paradoxical kinesia in rats exhibiting motor deficits after sub-chronic haloperidol, as we previously showed after acute haloperidol treatment.

## Introduction

Resulting from a pathophysiologic loss or degeneration of dopaminergic neurons in the substantia nigra pars compacta and the development of neuronal Lewy bodies, Parkinson’s disease (PD) is characterized by both motor and non-motor symptoms. Among the motor symptoms, PD patients classically display rest tremor, rigidity, bradykinesia, and stooping posture. Drugs aimed at increasing DA activity are prescribed to PD patients. In contrast, antipsychotics belonging to the first generation of antipsychotic drugs (McCue et al. 2006), such as haloperidol, block and reduce the effects of DA and therefore, even in therapeutic doses, can cause severe extra-pyramidal side effects resembling PD symptoms (Lockwood and Remington 2015). These side effects may differ after acute and chronic exposure. For instance, acute haloperidol treatment can cause Parkinsonism, which is reversible on cessation of treatment. Long-term haloperidol treatment can cause tardive dyskinesia, a debilitating motor side effect which is rarely reversible (Lockwood and Remington 2015).

In rats, acute haloperidol induces an immobility state named catalepsy that models akinesia in humans, and that has been well characterized as an animal model of acute Parkinsonism (Sanberg 1980). Recently, we showed that haloperidol-induced catalepsy in rats can be ameliorated by an emotionally and motivationally relevant appetitive auditory stimulus, named 50-kHz ultrasonic vocalizations (USV; Tonelli et al. 2018a). In general, USV are a prominent component of the behavioral repertoire displayed by rats and serve important communicative functions as situation-dependent socio-affective signals (Brudzynski 2013; Wöhr and Schwarting 2013). Specifically, 50-kHz USV are typical for social situations with positive valence, like juvenile play (Knutson et al. 1988) or sexual encounters (Barfield and Geyer 1972), and are believed to reflect a positive affective state (“rat laughter”; Panksepp 2005). As repeatedly shown by means of our 50-kHz USV radial arm maze playback paradigm, appetitive 50-kHz USV lead to social approach behavior in the recipient (Wöhr and Schwarting 2007; Engelhardt et al. 2017).

The fact that 50-kHz USV ameliorate haloperidol-induced catalepsy, in that they lead to a temporary state of efficient mobility induced by an emotional/motivational relevant auditory external trigger in Parkinsonian rats, was interpreted as an animal model of paradoxical kinesia. This term was suggested by Souques (1921) who described, for the first time, a brief improvement of motor performance in response to intense, alerting, or arousing stimuli, in akinetic/bradykinetic PD patients. Paradoxical kinesia suggests that patients, despite having relatively intact motor programs, present difficulties in accessing them without an external trigger, such as a loud noise or an important visual cue (Bloxham et al. 1987; Glickstein and Stein 1991; Jankovic 2008; Melo-Thomas and Thomas 2015). It has been also suggested that external triggers may activate alternatives motor pathways in order to induce paradoxical kinesia thereby temporarily improving motor deficits. In that way, we demonstrated that the inferior colliculus (IC), a midbrain structure which is not only implicated in auditory processing but has also motor outputs (Casseday and Covey 1996), can be part of some alternative pathway in order to produce paradoxical kinesia (Melo et al. 2010; Iacopucci et al. 2012; Tostes et al. 2013; Medeiros et al. 2014; Melo-Thomas and Thomas 2015; Tonelli et al. 2018b).

Although haloperidol-induced catalepsy in rats has a valuable place in the discovery of symptomatic drugs for PD and models human akinesia, it produces an acute motor impairment. In a condition like PD where patients suffer from chronic motor impairments, typically slowness of movement, it is paramount to assess the long-term symptom relief in an animal model of Parkinsonism. In line with this, sub-chronic haloperidol treatment significantly reduces locomotor activity, resembling a bradykinesia state in rats (Amato et al. 2011, 2018). The present study addressed the question whether 50-kHz USV playback ameliorates psychomotor deficits induced by haloperidol in a sub-chronic dosing regimen implemented by drug administration via osmotic mini-pumps.

## Material and methods

### Subjects

*N* = 30 male Wistar rats (Charles River Deutschland), weighing between 200 and 250 g at the beginning of the experiment, were used. Upon delivery, the rats were grouped in cages of five each for at least 7 days and after mini-pump implantation, they were housed in pairs. All rats had free access to food and water and were kept in a 12:12-h dark/light cycle (lights on at 07:00 am) and with a room temperature of 22 ± 2 °C and humidity of 55 ± 5% under a regular 12 h:12 h light/dark cycle (lights on from 7:00 a.m.). All experiments were conducted in accordance with the Animal Protection Law of the Federal Republic of Germany and the European Communities Council Directive of 24 November 1986 (86/609/EEC). All experimental procedures were approved by the ethical committee of the local government (Regierungspräsidium Gießen, Germany, TVA Nr: 124-2014).

### General overview

**Fig. 1:**
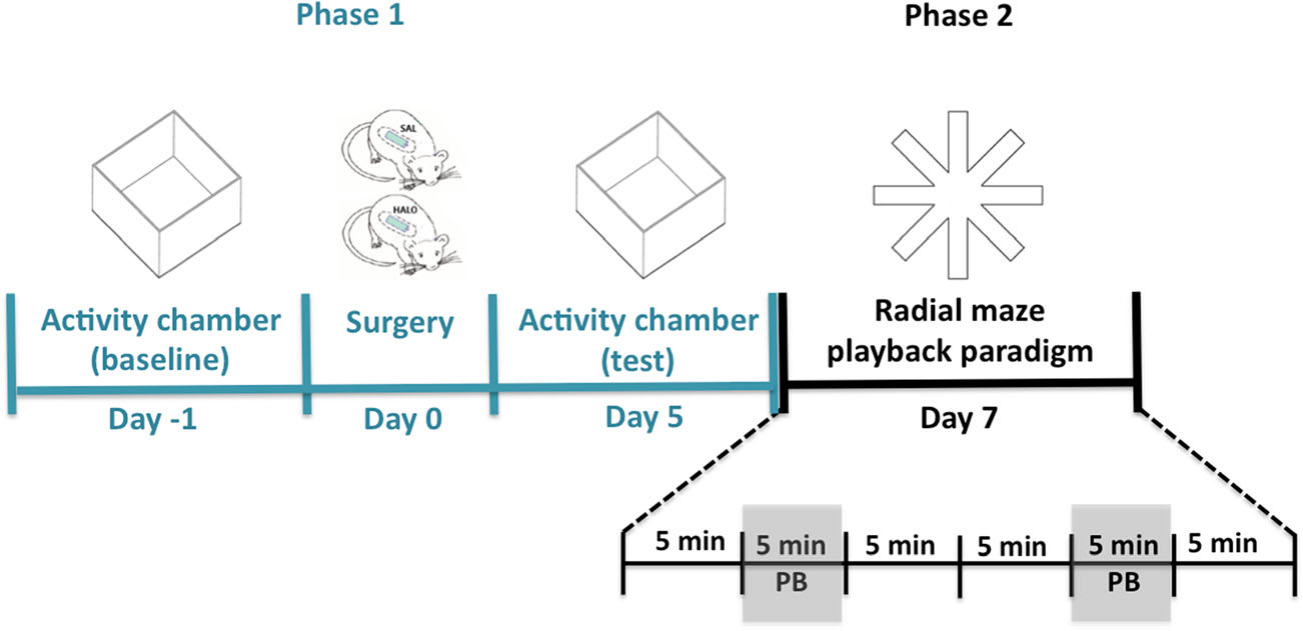
Experimental design of behavioral tests to assess the effect of 50-kHz USV on psychomotor deficits induced by sub-chronic haloperidol treatment. During phase 1, behavioral activity was monitored in an activity chamber 1 day before (baseline) and 5 days after osmotic mini-pump implantation, which allows continuous subcutaneous administration of saline or haloperidol (0.5 mg/kg/day). The radial maze playback paradigm was performed on day 7 (after osmotic mini-pump implantation). Gray bars indicate that 50-kHz USV playback (PB) was presented during 5 min

The experiment consisted of two phases: In phase 1, exploratory behaviors were assessed in an activity chamber before and 5 days after mini-pump surgery in order to investigate whether sub-chronic haloperidol treatment induced the expected psychomotor impairments. In phase 2, we investigated whether 50-kHz USV playback could overcome these psychomotor impairments by assessing exploratory behaviors and approach behavior towards the sound source in the 50-kHz USV radial maze playback paradigm (see Fig. 1).

#### Mini-pump implantation and drug delivery

Osmotic pumps containing saline (*N* = 15) or haloperidol (*N* = 15) were implanted under anesthesia with 3.5% isoflurane (Baxter Deutschland GmbH, Germany) in oxygen. A 2.0-cm-wide incision was made in each animal’s lower back. Preloaded mini-pumps were wiped with 70% isopropyl alcohol, and positioned between the scapulae, with the flow moderator directed away from the skin incision, which was closed using sterile 9 mm surgical staples. An antiseptic aluminum formulation (Aluspray® 210 ML) was used to prevent local infections (Samaha et al. 2007; Amato et al. 2011, 2018).

After surgery, haloperidol (0.5 mg/kg/day; Amato et al. 2011) or saline were delivered over a period of 7 days (sub-chronic treatment) via the osmotic mini-pump. According to previous studies, we used a representative time schedule of treatment that yielded psychomotor impairment in rats (Amato et al. 2011). After surgery, the rats were kept in pairs with ad libitum access to food and water.

#### Experimental setups and behavioral tests

##### Activity chamber

Behavioral activity was recorded during 15 min using an activity chamber (40 × 40 × 40 cm) under red light (28 lx) according to a previously established protocol (Natusch and Schwarting 2010). Testing occurred 1 day before (baseline) and 5 days after surgery for osmotic mini-pump implantation. At the start of each test session, the given rat was placed into the center of the chamber and the test session started immediately. Distance traveled (cm) and number of rearing behavior were assessed on-line using an infrared sensor beam system mounted 2.5 cm above the floor of the arena (TruScan™, Photobeam Sensor-E63-22, Coulbourn Instruments, PA, USA). The chamber was cleaned (0.1% acetic acid solution) and dried thoroughly between testing sessions.

##### 50-kHz USV radial maze playback paradigm

On the seventh day after surgery, the 50-kHz USV radial maze paradigm was conducted (Seffer et al. 2014). Playback of acoustic stimuli was performed under dim red light (∼ 10 lx) on a radial eight-arm maze made of black plastic, elevated 52 cm above the floor. The arms (9.8 × 40.5 cm) extended radially from a central platform (diameter 24 cm). Acoustic stimuli were presented through an ultrasonic loudspeaker (ScanSpeak, Avisoft Bioacoustics, Berlin, Germany) using an external sound card with a sampling rate of 192 kHz (Fire Wire Audio Capture FA-101, Edirol, London, UK). The loudspeaker, which had a frequency range of 1–120 kHz with a relatively flat frequency response (± 12 dB) between 15 and 80 kHz, was placed 20 cm away from the end of one arm. An additional, but inactive ultrasonic loudspeaker was arranged symmetrically at the opposite side as a visual control. Rats were tested in a testing room with no experimenter or other rats present. Behavioral tests were conducted between 8 and 18 h. Before each test, behavioral equipment was cleaned (0.1% acetic acid solution) and dried.

Rats were exposed to two playback presentations of 50-kHz USV with a sampling rate of 192 kHz in 16 bit format at ∼ 69 dB (measured from a distance of 40 cm). The 50-kHz USV had been recorded from an adult male Wistar rat during exploration of a cage containing scents from a cage mate after being separated from it (for setting and recording, see Wöhr et al. 2008). The acoustic stimulus material was composed of a sequence lasting 3.5 s, which was presented in a loop. Each sequence contained 13 50-kHz calls (total calling time 0.90s), with 10 of them being frequency-modulated, i.e., characterized by an up-and-down in frequency, and 3 flat, i.e., with constant frequency. Behavior was monitored by a video camera (Panasonic WV-BP 330/GE, Hamburg, Germany) from about 150 cm above the radial maze, which fed into an external multimedia hard drive (ScreenPlay Pro HD, Iomega, San Diego, CA, USA).

Rats were placed individually onto the central platform of the radial maze with their body axis at an angle of 90° to the two ultrasonic loudspeakers. After an initial habituation phase of 5 min where no acoustic stimulus was presented, the rats were exposed to 50-kHz USV playback for 5 min followed by an additional 5 min period without any sound presentation. This procedure was repeated one more time. The video recordings were analyzed by an automated video tracking system (EthoVision XT, Noldus Information Technology) which scored the behaviors for the following standard parameters: (I) number of entries into the three arms proximal to or distal from the active ultrasonic loudspeaker; (II) the time spent on proximal and distal arms; (III) total distance traveled. An arm entry was counted when all four paws were within the arm.

##### Statistical analysis

Behavioral activity assessed in the activity chamber was analyzed using two-way ANOVAs for repeated measurements with the between-subject factor DRUG and the within-subject factor DAY, followed by unpaired *t* tests when appropriate. Psychomotor activity assessed in the playback paradigm was analyzed using two-way ANOVAs for repeated measurements with the between-subject factor DRUG and the within-subject factor MINUTE followed by unpaired *t* tests when appropriate. Arm entries and time spent in a given arm were analyzed using two-way ANOVAs for repeated measurements with the within-subject factor ARM preference for proximal versus distal arms and the within-subject factor MINUTE, followed by paired *t* tests when appropriate. For comparing haloperidol treated rats and saline controls, two-way ANOVAs for repeated measurements with the between-subject factor DRUG and the within-subject factor ARM preference were applied, followed by unpaired *t* tests when appropriate. Separate analyses were conducted for the first and the second playback phase. Statistical analyses were performed using IBM SPSS software Statistics 22. A *p* value of < 0.05 was considered statistically significant. Four rats (2 per group) had to be excluded from all statistical analyses for technical reasons (data loss).

## Results

### Reduced behavioral activity in rats exposed to sub-chronic haloperidol treatment in the activity chamber

**Fig. 2:**
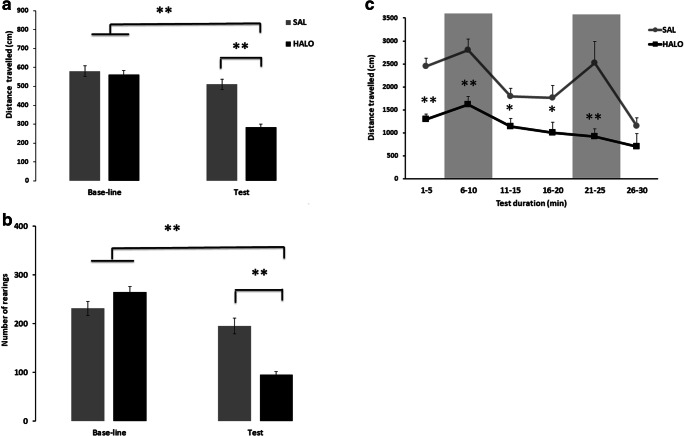
Sub-chronic haloperidol treatment impaired psychomotor function as assessed in the activity box or radial maze USV paradigm. Psychomotor activity was assessed during 5 min by **a** total distance traveled and **b** rearing numbers in the activity box or **c** total distance traveled in the radial maze USV paradigm before (baseline) and after (test) chronic haloperidol administration (0.5 mg/kg/day, IP) or saline. Bars represent means ± SEM. *P < 0.05 and **P < 0.001 as compared to saline (repeated measures ANOVA)

Sub-chronic haloperidol treatment affected psychomotor activity in the activity chamber (DRUG: *F*_1,24_ = 19.483, *p* < 0.001; DAY: *F*_1,24_ = 90.111, *p* < 0.001; DRUG × DAY: *F*_1,24_ = 36.032, *p* < 0.001; Fig. 2a). While psychomotor activity did not differ between treatment during baseline conditions before surgery (*t*_24_ = 0.751, *p* = 0.460), distance traveled was lower in haloperidol treated rats, as compared to saline controls (*t*_24_ = 7.470, *p* < 0.001). A similar result pattern was obtained for rearing behavior (DRUG: *F*_1,24_ = 4.787, *p* = 0.039; DAY: *F*_1,24_ = 140.793, *p* < 0.001; DRUG × DAY: *F*_1,24_ = 57.140, *p* < 0.001; Fig. 2b). Again, no difference was evident under baseline conditions before surgery (*t*_24_ = 1.783, *p* = 0.087). After surgery, however, haloperidol treated rats displayed fewer rearings than saline controls (*t*_24_ = 5.499, *p* < 0.001). Together, this indicates that sub-chronic haloperidol treatment led to the expected psychomotor deficits.

### Reduced psychomotor activity in rats exposed to sub-chronic haloperidol treatment during 50-kHz USV playback

Evidence for psychomotor deficits was also obtained in the 50-kHz USV radial maze playback paradigm, with psychomotor activity being low from the very beginning in haloperidol treated rats (Fig. 2c). Specifically, during the first playback trial, distance traveled was lower in haloperidol treated rats, as compared to saline controls, irrespective of test phase, i.e., before, during, and after 50-kHz USV playback. Still, 50-kHz playback enhanced locomotion and psychomotor activity was higher during than before or after 50-kHz USV playback with the increase in psychomotor activity evoked by 50-kHz USV playback evident in haloperidol treated rats and saline controls (DRUG: *F*_1,24_ = 32.531, *p* < 0.001; MINUTE: *F*_14,336_ = 4.310, *p* < 0.001; DRUG × MINUTE: *F*_14,336_ = 1.264, *p* = 0.228).

During the second playback presentation, distance traveled was again lower in haloperidol treated rats than in saline controls, yet psychomotor activity during 50-kHz USV playback was affected by haloperidol treatment, since the increase in psychomotor activity evoked by 50-kHz USV playback was evident in saline controls but not haloperidol treated rats (DRUG: *F*_1,24_ = 11.646, *p* = 0.002; MINUTE: *F*_14,336_ = 1.277, *p* = 0.219; DRUG × MINUTE: *F*_14,336_ = 1.834, *p* = 0.033).

### Playback of 50-kHz USV playback leads to social approach behavior in saline controls

**Fig. 3:**
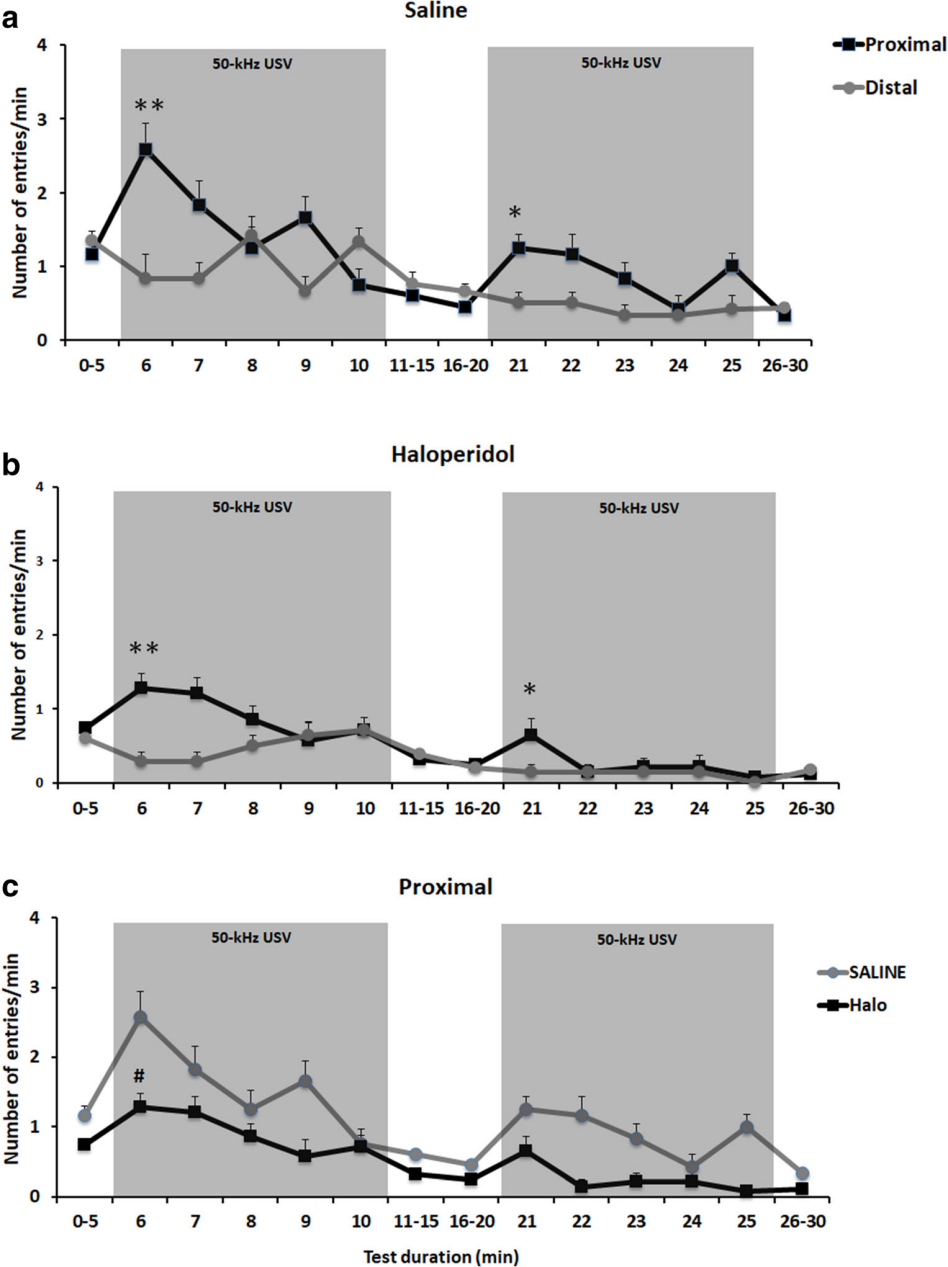
Psychomotor impairment induced by chronic haloperidol treatment ameliorates during playback of acoustic stimuli as assessed by number of entries on radial maze arms. Spontaneous locomotion measured by numbers of entries on arms distal and proximal to the ultrasonic loudspeaker in response to 2 presentations of 50-kHz USV, in rats receiving sub-chronic saline (**a**) or haloperidol treatment (**b**). **c** Although sub-chronic haloperidol treatment induces a psychomotor impairment, an increase in psychomotor activity was evoked during 50-kHz USV playback presentations in both haloperidol treated rats and saline controls. Dots represent means ± SEM. *P < 0.05 and **P < 0.005 as compared to before PB; ^♯^*P* < 0.05 as compared to saline proximal arms (independent samples *t* test)

**Fig. 4:**
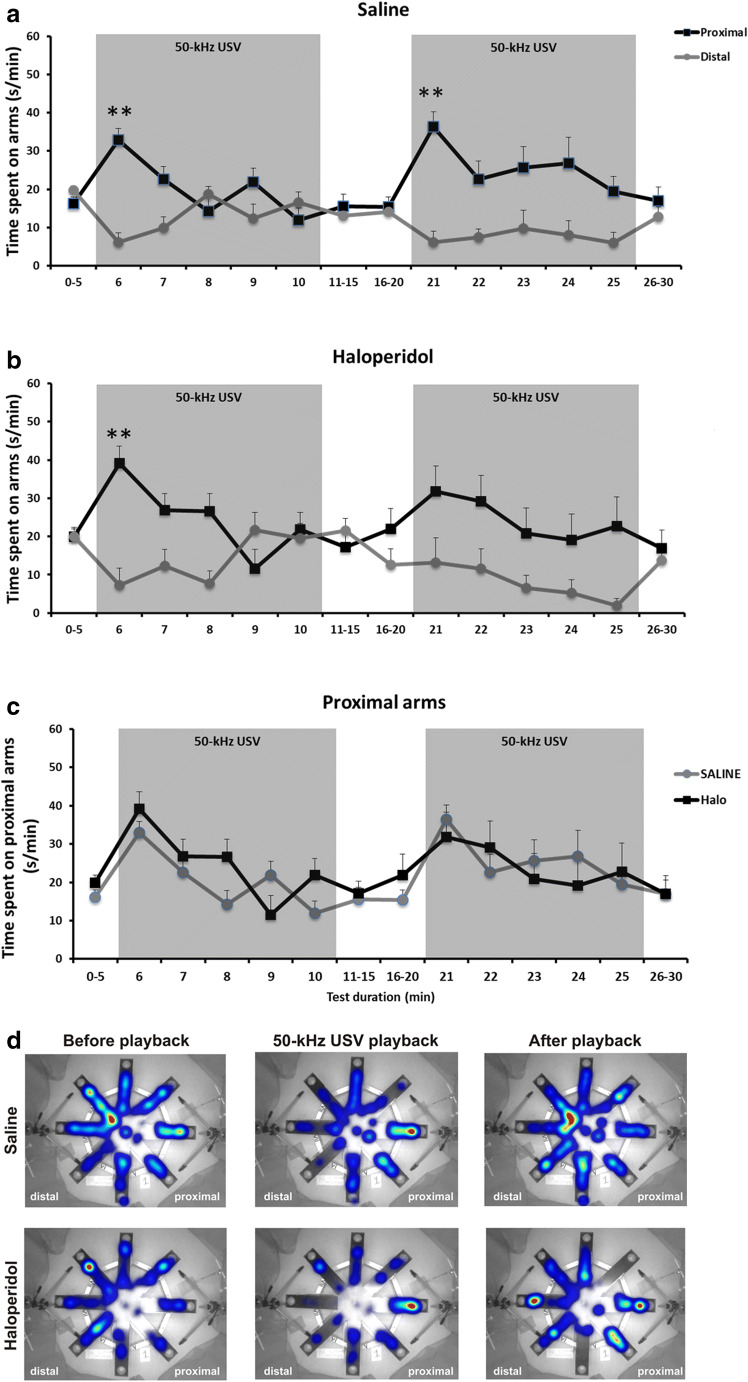
Psychomotor impairment induced by sub-chronic haloperidol treatment ameliorates during playback of acoustic stimuli as assessed by time spent on radial maze arms. Time spent on arms distal and proximal to the ultrasonic loudspeaker in response to 2 presentations of 50-kHz USV (gray boxes), in rats receiving sub-chronic saline (**a**) or haloperidol treatment (**b**). **c** Although sub-chronic haloperidol treatment induces a psychomotor impairment, approaching behavior towards the sound source was evoked by 50-kHz USV playback in both haloperidol treated rats and saline controls. **d** Heat maps depict corresponding spatial distributions of exploratory behavior 1 min before, during, and after PB. Dots represent means ± SEM. **P < 0.005 as compared to before and after PB (independent samples *t* test)

In saline controls, the increase in psychomotor activity evoked by 50-kHz USV playback was directed towards the sound source. Social approach behavior induced by 50-kHz USV was evident during the first and second playback trial and reflected in a preference for proximal arms exclusively during 50-kHz USV playback. Specifically, in the first playback presentation, social approach behavior evoked by 50-kHz USV playback was reflected in a time-dependent preference for proximal over distal arms at the level of arm entries (ARM: *F*_1,154_ = 2.087, *p* = 0.176; MINUTE: *F*_14,154_ = 4.630, *p* < 0.001; ARM × MINUTE: *F*_14,154_ = 3.064, *p* < 0.001; Fig. 3a) and the time spent on arms (ARM: *F*_1,154_ = 2.342, *p* = 0.154; MINUTE: *F*_14,154_ = 1.703, *p* = 0.060; ARM × MINUTE: *F*_14,154_ = 2.439, *p* = 0.004; Fig. 4a). Saline controls displayed more proximal than distal arm entries during 50-kHz USV playback (*t*_11_ = 3.317, *p* = 0.007), particularly during the first minute of presentation (*t*_11_ = 3.251, *p* = 0.008). They further spent more time on proximal than distal arms during 50-kHz USV playback (*t*_11_ = 2.976, *p* = 0.013), again particularly during the first minute of presentation (*t*_11_ = 6.190, *p* < 0.001).

### Repeated 50-kHz USV playback leads to less prominent social approach behavior in saline controls

During the second playback presentation, a weaker response was evident in saline controls, particularly at the level of arm entries (ARM: *F*_1,154_ = 1.099, *p* = 0.317; MINUTE: *F*_14,154_ = 2.545, *p* = 0.003; ARM × MINUTE: *F*_14,154_ = 2.389, *p* = 0.005; Fig. 3a) but less so for the time spent on arms (ARM: *F*_1,154_ = 27.730, *p* < 0.001; MINUTE: *F*_14,154_ = 0.966, *p* = 0.491; ARM × MINUTE: *F*_14,154_ = 1.527, *p* = 0.107; Fig. 4a). Consistent with the first playback presentation, saline controls displayed more proximal than distal arm entries during 50-kHz USV playback (*t*_11_ = 4.584, *p* = 0.001), particularly during the first minute of presentation (*t*_11_ = 3.458, *p* = 0.005). They further spent more time on proximal than distal arms (*t*_11_ = 3.959, *p* = 0.002), again particularly during the first minute of presentation (*t*_11_ = 4.897, *p* < 0.001).

### Playback of 50-kHz USV leads to social approach behavior in rats exposed to sub-chronic haloperidol treatment

Although the increase in psychomotor activity evoked by 50-kHz USV playback was weaker in haloperidol treated rats, it was still directed towards the sound source. As in saline controls, social approach behavior induced by 50-kHz USV was evident during the first playback presentation and reflected in a preference for proximal arms exclusively during 50-kHz USV playback. Specifically, during the first playback presentation, social approach behavior evoked by 50-kHz USV playback was reflected in a time-dependent preference for proximal over distal arms at the level of arm entries (ARM: *F*_1,182_ = 8.876, *p* = 0.011; MINUTE: *F*_14,182_ = 3.442, *p* < 0.001; ARM × MINUTE: *F*_14,182_ = 3.069, *p* < 0.001; Fig. 3b) and the time spent on arms (ARM: *F*_1,182_ = 0.546, *p* = 0.473; MINUTE: *F*_14,182_ = 1.662, *p* = 0.067; ARM × MINUTE: *F*_14,182_ = 3.034, *p* < 0.001; Fig. 4b). Similar to saline controls, haloperidol treated rats displayed more proximal than distal arm entries during 50-kHz USV playback (*t*_13_ = 4.296, *p* = 0.001), particularly during the first minute of presentation (*t*_13_ = 4.266, *p* = 0.001). They further spent more time on proximal than distal arms during 50-kHz USV playback (*t*_13_ = 3.098, *p* = 0.008), again particularly during the first minute of presentation (*t*_13_ = 3.866, *p* = 0.002).

### Repeated 50-kHz USV playback leads to less prominent social approach behavior in rats exposed to sub-chronic haloperidol treatment

Consistent with the lack of an increase in psychomotor activity evoked during the second playback presentation, a clearly weaker response was evident in haloperidol treated rats, both at the level of arm entries (ARM: *F*_1,182_ = 2.519, *p* = 0.136; MINUTE: *F*_1,182_ = 1.161, *p* = 0.309; ARM × MINUTE: *F*_1,182_ = 1.469, *p* = 0.126) and the time spent on arms (ARM: *F*_1,182_ = 3.661, *p* = 0.078; MINUTE: *F*_14,182_ = 1.654, *p* = 0.069; ARM × MINUTE: *F*_14,182_ = 0.625, *p* = 0.842). Despite the overall weaker response, however, haloperidol treated rats still displayed more proximal than distal arm entries during 50-kHz USV playback (*t*_13_ = 2.687, *p* = 0.019), again particularly during the first minute of presentation (*t*_13_ = 2.463, *p* = 0.029). They further spent more time on proximal than distal arms (*t*_13_ = 2.886, *p* = 0.013), although not reaching statistical significance in the first minute of presentation (*t*_13_ = 1.537, *p* = 0.148).

Of note, no preference for proximal arms was evident before or after 50-kHz USV playback and number of proximal and distal arm entries as well as the time spent therein did not differ (all *p* values > 0.100), except for more distal than proximal arm entries before the second playback in saline controls (*t*_11_ = 4.584, *p* = 0.001).

### Social approach behavior evoked by 50-kHz USV playback is similar in rats exposed to sub-chronic haloperidol treatment and saline controls

When comparing haloperidol treated rats and saline controls during the first playback presentation, proximal arm entries were consistently lower in haloperidol treated rats, as compared to saline controls, irrespective of test stage, i.e., before, during, and after 50-kHz USV playback, with differences being particularly prominent during 50-kHz USV playback (DRUG: *F*_1,24_ = 31.009, *p* < 0.001; MINUTE: *F*_14,336_ = 8.328, *p* < 0.001; DRUG × MINUTE: *F*_14,336_ = 1.850, *p* = 0.031; Fig. 3c), reflecting psychomotor deficits in haloperidol treated rats. Despite psychomotor deficits, however, the time spent proximal did not differ between haloperidol treated rats and saline controls during the first playback presentation, with both displaying a robust increase in the time spent proximal during 50-kHz USV playback (DRUG: *F*_1,24_ = 2.048, *p* = 0.165; MINUTE: *F*_14,336_ = 3.424, *p* < 0.001; DRUG × MINUTE: *F*_14,336_ = 1.394, *p* = 0.154; Fig. 4c), indicating intact social approach behavior in haloperidol treated rats. During the second playback presentation, a similar result pattern characterized by psychomotor deficits (DRUG: *F*_1,24_ = 35.200, *p* < 0.001; MINUTE: *F*_14,336_ = 4.174, *p* < 0.001; DRUG × MINUTE: *F*_14,336_ = 2.025, *p* = 0.031; Fig. 3c) but intact yet weaker social approach behavior (DRUG: *F*_1,24_ = 0.160, *p* = 0.693; MINUTE: *F*_14,336_ = 1.640, *p* = 0.067; DRUG × MINUTE: *F*_14,336_ = 0.687, *p* = 0.787; Fig. 4c) was evident.

## Discussion

Psychomotor deficits were investigated in the present study using an activity box, commonly applied to assess the action of drugs or other manipulations that promote psychomotor abnormalities (bradykinesia or hyperlocomotion; Seibenhener and Wooten 2015). We used this test in order to assess rat psychomotor function through horizontal (distance traveled) and vertical (rearing) exploratory behaviors. Sub-chronic haloperidol treatment led to psychomotor deficits since the distance traveled and number of rearing behavior were clearly reduced as compared to the saline control group or to baseline. Evidence for psychomotor deficits was also obtained in the 50-kHz USV radial maze playback paradigm, with distance traveled being lower in haloperidol treated rats. These data are in line with previous findings showing that sub-chronic haloperidol treatment by osmotic mini-pump, at the same dose regimen as used here, reduced locomotor activity. An increase in activity in those animals could also be induced by different stimuli than that used in our study, namely novelty, food, tail-pinch, or an amphetamine challenge (Amato et al. 2011, 2018; Groos et al. 2019).

The psychomotor impairments shown in the present study can be interpreted as a state of bradykinesia induced by sub-chronic haloperidol treatment. Here, it is worth to define akinesia and bradykinesia. These are interrelated terms and characterize a negative symptom of Parkinsonism, namely the reduction of motor activity. Literally translated, “akinesia” means immobility. However, except in the case of extremely severe states, Parkinsonian humans as well as animals are not totally unable to move, so that this term usually refers to the impaired ability to initiate movements. “Bradykinesia” points to the fact that movements in Parkinsonian individuals are slower than observed in healthy controls (Sedelis et al. 2001). In rodents, acute haloperidol administration (systemic or intrastriatal) induces a behavioral state known as catalepsy in which the animals are unable to correct externally imposed postures (Sanberg 1980). For this reason, acute haloperidol-induced catalepsy models akinesia (Castagne et al. 2009; Sanberg et al. 1988; Wadenberg 1996) in rats, whereas, as shown at the present study, sub-chronic haloperidol treatment reduced exploratory behavior in rats, which resembles bradykinesia. Interestingly, acute and sub-chronic haloperidol treatments lead not only to different behavioral outcomes but also to different brain changes. The cataleptic effects induced by acute haloperidol treatment are mediated by blockade of postsynaptic DA D2 receptors in the striatum (Sanberg 1980), where they are mainly located on GABAergic projection neurons and cholinergic interneurons (Johnson et al. 2014; Kharkwal et al. 2016). On the other side, Amato et al. (2011), using the same injection procedure (osmotic mini-pump) as used in the present study, showed that after sub-chronic haloperidol treatment, medial prefrontal cortex (mPFC), but not nucleus accumbens and caudate putamen (striatum) extracellular DA levels were decreased. Therefore, bradykinesia observed in the present study could be assigned to a decreased extracellular DA level in the mPFC.

The current study shows for the first time that these psychomotor impairments induced by sub-chronic administration of haloperidol can be ameliorated during playback of 50-kHz USV, i.e., a relevant and motivational auditory stimuli. Although the increase in psychomotor activity evoked by 50-kHz USV playback appeared weaker in haloperidol treated rats due to their overall reduction in distance traveled, it was still directed towards the sound source. Haloperidol treated rats showed a clear social approach behavior during 50-kHz USV playback presentation reflecting a clear preference for proximal arms exclusively during playback. Particularly, in the first playback presentation, social approach behavior evoked by 50-kHz USV playback was reflected in the increased number of entries and time-dependent preference for proximal over distal arms.

Thus the present data suggest that 50-kHz USV playback, as an emotional and motivationally relevant appetitive auditory stimulus, can overcome the psychomotor impairments induced by sub-chronic haloperidol treatment in bradykinetic rats. This data strengths our previous findings showing that playback of 50-kHz USV reduce acute haloperidol-induced catalepsy in rats, providing a completely new approach to study mechanisms of paradoxical kinesia induced by appetitive auditory stimuli (Tonelli et al. 2018a). Paradoxical kinesia is the sudden transient ability of a patient with PD to perform a task he or she was previously unable to perform (Glickstein and Stein 1991). Although paradoxical kinesia usually occurs in stressful situations, it can be triggered by visual and/or auditory stimuli as well. Sometimes, this exciting condition reaches such a degree that a previously incapacitated patient cannot be distinguished from perfectly healthy person. Besides, paradoxical kinesia vanishes as suddenly and rapidly as it appears, i.e., its duration may vary from a few seconds to minutes (Sacks 1973) in patients. Similarly, in the present study, the time spent proximal did not differ between haloperidol treated rats and saline controls during the playback presentation (see Fig. 3c), with both displaying a robust increase in the time spent proximal during 50-kHz USV playback. Still, the psychomotor improvement shown by sub-chronic haloperidol treated rats vanished at the end of the 50-kHz USV playback presentation, i.e., no preference for proximal arms was evident after 50-kHz USV playback. These data indicate that 50-kHz USV playback induces paradoxical kinesia in rats exhibiting psychomotor deficits after sub-chronic haloperidol.

Together our previous and present data carry important contributions to understand the phenomenon of paradoxical kinesia induced by appetitive auditory stimuli. The same auditory stimuli (50-kHz USV) compensated efficiently psychomotor deficits induced by both acute (catalepsy; Tonelli et al. 2018a) or sub-chronic (bradykinesia; present data) haloperidol treatment, although these psychomotor deficits are ascribed to different neural mechanisms (Sanberg 1980; Amato et al. 2011, 2018). This is in accordance with previous data suggesting that specific external stimuli can lead to paradoxical kinesia by “energizing” relevant action systems in the brain (Ballanger et al. 2006), which are otherwise insufficiently activated. Here, 50-kHz USV may be especially suitable, since they have motivational properties, i.e., approach-inducing quality for the recipient (Wöhr and Schwarting 2007). Indeed, it is assumed that PD patients may have intact motor programs but have difficulty accessing them without external sensory stimulation (Jankovic 2008; Clark et al. 2009) and paradoxical kinesia might work to improve motion by activation of basal ganglia reserves or via alternative pathways to somehow improve motion (Glickstein and Stein 1991). However, the nature of this alternative pathway is unknown. Using the animal models of paradoxical kinesia established by our group, we have been searching for the presumed alternative pathway underlying this phenomenon. We have proposed that external auditory stimulation activating the IC may trigger motor circuits even when striatal dopamine transmission is impaired during neuroleptic-induced catalepsy (Medeiros et al. 2014; Melo et al. 2010; Tostes et al. 2013). The IC is a relay station integrating descending and ascending auditory information; the latter is known as the main auditory thalamic relay, projecting from the IC to the medial geniculate nucleus and thus to the auditory cortex (Cappe et al. 2009). It is possible that an auditory-motor integration takes place at cortical levels since anatomical studies have shown that the output of the auditory cortex reaches wide areas of the prefrontal cortex (Jones and Powell 1970; Fuster 1989; Pandya and Kuypers 1969; Van Eden et al. 1992). Here, it is important to highlight that the IC may contribute to this cortical auditory-motor integration since electrical stimulation of the IC produced a long-lasting increase in the levels of DA in the frontal cortex (Cuadra et al. 2000). Thus we can speculate that the auditory stimulation used here (50-kHz USV) as processed by the IC may increase DA levels in the frontal cortex, compensating dopaminergic extracellular reduced levels induced by sub-chronic haloperidol treatment and accordingly, improve bradykinesia.

Additional evidence pointing to the IC as part of the neural substrate involved in paradoxical kinesia has already been provided by our group. We demonstrated that intracollicular microinjections of glutamatergic and GABAergic drugs modulate haloperidol-induced catalepsy (Melo et al. 2010; Tostes et al. 2013). Beyond that, catalepsy induced by haloperidol can be reduced by both low and high frequency (Engelhardt et al. 2018; Melo-Thomas and Thomas 2015) electrical deep brain stimulation in the IC, representing an animal model of paradoxical kinesia. These studies provided evidence that the IC can serve as an alternative, non-conventional deep brain stimulation target to improve symptoms of motor disorders. It remains to be tested whether intracollicular deep brain stimulation can ameliorate bradykinesia induced by sub-chronic haloperidol treatment, as shown in the present study.

Another interesting point that remains to be clarified is whether the sensory (auditory)-motor transformation observed in the present study may encompass motivational aspects of the trigger. We had previously found that 50-kHz USV, as used here, lead to phasic DA release in the nucleus accumbens (Willuhn et al. 2014), which probably underlies its approach-eliciting effects in intact animals. It remains to be shown, however, whether this is the case in the present study since Amato et al. (2011) showed that after sub-chronic haloperidol treatment, as used here, the DA extracellular level in the nucleus accumbens was not affected and DA responses to appetitive stimulation (food consumption) were largely preserved. Besides that, it has been suggested that paradoxical kinesia is not mediated by actions on striatal DA (Keefe et al. 1989). However, the role of an emotional aspect of the trigger in guiding paradoxical kinesia cannot be excluded. The strong emotional/motivational aspect carried by 50-kHz USV can induce paradoxical kinesia through activation of alternative pathways. Again, here the IC may provide a critical site since it is part of the brain aversive system (Brandão et al. 1993; Melo and Brandão 1995) and sustains the development of emotional responses to acoustic stimuli (Ledoux et al. 1984, 1986). Thus, the emotional aspects of auditory stimuli as processed in the IC can lead to some kind of “energizing” in the motor system in order to generate a proper behavioral response to such emotional/motivational relevant stimuli. This also is in accordance with the proposition that paradoxical kinesia might be also mediated by alternative routes by-passing the basal ganglia (Glickstein and Stein 1991; Jankovic 2008; Melo et al. 2010; Melo-Thomas and Thomas 2015).

In conclusion, the present data provide evidence that 50-kHz USV playback induces paradoxical kinesia in rats exhibiting motor deficits after sub-chronic haloperidol, as we previously showed after acute haloperidol treatment which induces a transient state of catalepsy. It remains to be clarified the exact neural mechanism underlying paradoxical kinesia and how the auditory stimulus triggers this phenomenon.
